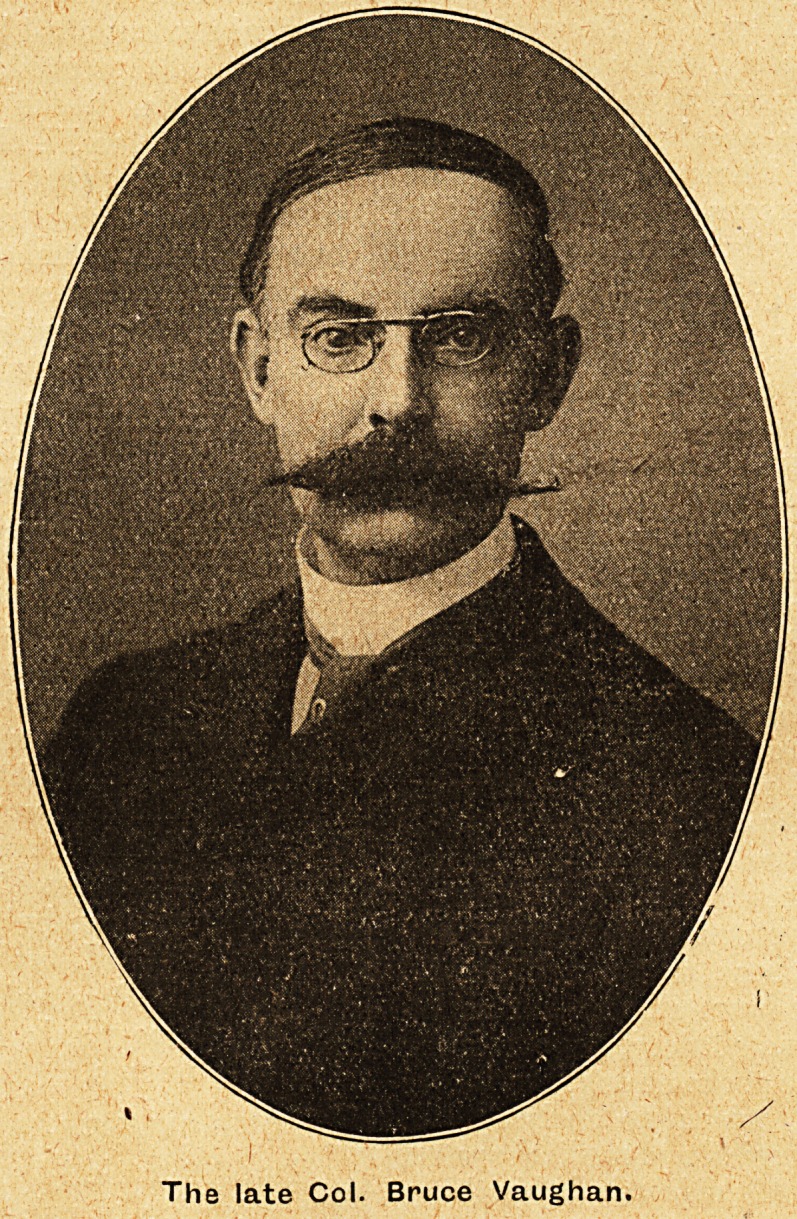# Hospital and Institutional News

**Published:** 1919-06-28

**Authors:** 


### HOSPITAL AND INSTITUTIONAL NEWS.

### THE DEATH OF COLONEL BRUCE VAUGHAN.

In our last issue we gave an idea of the work and results secured by Colonel Bruce Vaughan, the well-known chairman of the House Committee of King Edward VII. Hospital, Cardiff, whose work made him and the hospital which he helped to reconstruct famous throughout the country. We may add to-day that the equipment of the hospital is remarkable for its comprehensiveness; provision has been made for four large operating-theatres, and elaborately equipped and fully appointed electrical massage, hydro-therapeutic, and pathological departments, whilst the practical aspects have not been neglected; the new kitchen now just completed is probably one of the finest in the kingdom. Apart from the hospital itself, Colonel Vaughan has brought several auxiliary institutions into being, notably the Maternity Hospital in Glossop Road, the beautiful Lee Convalescent Home at Lavernock, and the Preliminary Training-School for Nurses at Anthony House. He was a man of large ideas, and the creation of the medical school, for which he did so much, will be a permanent and living memorial to his memory. Though illness interrupted the last period of his life, none but his intimates suspected that his great work was over. Fertile in suggestion, and persistent, he was content with nothing but the best, and the voluntary system has not produced a more inspiring example of its principles. With General Lee and Mr. Leonard Rea to aid him the great work was successfully launched and carried on, and it is a tribute to the voluntary system to produce such men, whose lives are dedicated to personal service. The wreath from the Board of Management was inscribed with the words, "In ever grateful memory of a good man and a great achievement. He whose care is for others forgets to be afraid." Because no difficulty daunted him, he was a true leader, who will live in the tradition that he created.

The late Col. Bruce Vaughan.

### THE ATTACK ON MR. CURRY.

Friends of Guy's Hospital and friends of Mr. W. J. Curry, the popular Clerk, will feel much sympathy, and a certain amount of astonishment, in respect of the recent outrage, a full account of which appears on another page. To the benevolent and malevolent alike it must be known that in a hospital, especially in a large one, there is always a certain amount of money at any given time—money that has come in in the form of revenue, and money for the payment of wages and the many daily incidental outgoings. In this a hospital does not differ greatly from a business house. Daylight attacks on banks and businesses are not unknown, but, as it happens, hospitals are comparatively immune from this class of outrage. Mr. Curry, we are sorry to say, was badly handled by his assailants, but is, fortunately, rapidly recovering from his injuries. On the whole, he and his hospital came off well.

### SIR JAMES CANTLIE IN THE PULPIT.

A surgeon and teacher of ambulance classes in great profusion, Sir James made a new departure on Hospital Sunday at St. Peter's, Vere Street, where he was announced to preach in the evening, on the invitation of Bishop Bury, Bishop of Northern and Central Europe. In the result he cannot be said to have preached, though he covered a wide field, for his remarks ranged from Huxley and Darwin, who as scientists were looked upon with distrust and alarm, Dean Stanley's insistence on Darwin's burial in Westminster Abbey at the foot of Sir Isaac Newton, and what Sir James holds to be "science's triumph in its fight against disease, which now has the blessing of God through His Church without which all is vanity." Mankind will have to continue the fight with disease, for so long as the world lasts every decade sees some disease curbed, checked, or rendered innocuous, whilst others arise, and there are always some diseases the presence of which continue virulent and destructive to mankind. Through applied science with the enforcement of hygiene in the widest meaning of the word typhus fever in London is practically extinct. The writer had the privilege of tracing the origin of and ending the last serious epidemic of typhus in London. Typhoid fever is now a rare disease. Small-pox, which one hundred years ago was so prevalent in this country, that every woman who was not pitted by small-pox was termed a fair woman, has altogether lost its terrors. Malaria, common a century ago in the south-east of England, and a little earlier still as prevalent a disease along the Banks of the Thames as in the East, is to-day practically a mere matter of history. Hydrophobia disappeared from England for fifteen years, and has only returned to-day brought back again by transgressors of the quarantine laws of our country. "These diseases have lost much of their terror, but would be with us in virulent form to-morrow, if we neglected the laws of sanitation." The speaker further dwelt on the recent virulence of influenza, which carried off many times more persons than death had claimed through war during the past five years.

### ENGLAND'S FRONT LINE OF DANGER.

Sir James reminded his hearers, lest they should grow self-confident, that the following diseases still maintain their hold in a virulent form: Measles, scarlet fever, whooping cough, rheumatic fever, and the great scourge of tuberculosis. These require to be dealt with forcibly and promptly by the new Public Health Department which took the place of the Local Government Board on the 25th instant. Muncipal, county and local authorities ought to combine to prevent the constantly increasing mortality through accidents in our streets. England's front line of danger for the working population arises from wounds received in the streets, manufactories, mines, and workshops. Sir James Cantlie strongly insisted that these evils, loss of life, suffering, and many other things could be reduced to a minimum and greatly remedied if every citizen, man and woman, made it his business to master first-aid work, that all might know how to help their neighbours in misfortune as a sacred duty, by supplying reliable voluntary aid. He spoke strongly in favour of the voluntary system, which he declared had yielded the most perfect hospital and medical system the world has ever seen. He elaborated this thesis with great fulness, practically covering most of the field of medicine and surgery and its subsidiary branches. He ended with an earnest appeal to his hearers to become Complete Samaritans by learning ambulance work and so fulfil Christ's teaching. His figures were out of date and therefore misleading. He gave, for instance, £168,000 in 1916 as the greatest amount ever given to the London hospitals in one year by King Edward's Hospital Fund. The fact is this Fund distributed £200,000 in the year 1918.

### PORTRAIT OF SIR A. BOWLBY.

It is proposed at St. Bartholomew's Hospital to present to Sir Anthony Bowlby a portrait of himself painted by a well-known artist, as a recognition not only of his services to the hospital from the staff of which he will shortly retire, but also as an appreciation which all Bart.'s men must feel for the great work that he has done in connection with the Army Medical Service during the war. The portrait will be hung in the Great Hall of the hospital, among those of his illustrious predecessors which already adorn it. Those in sympathy with this proposal are asked to forward subscriptions not exceeding two guineas to Mr. R. Cozens Bailey, The Warden's House, St. Bartholomew's Hospital, E.C. 1.

### OPHTHALMOLOGY IN THE MEDICAL CURRICULUM.

At its recently concluded session the General Medical Council adopted a recommendation of its Examination Committee, to the effect that every medical student shall be required to attend a course of practical instruction in ophthalmology of not less than ten weeks' duration, and must, before admission to the Final Examination, present a certificate showing that he has attended such a course regularly, and that his work in connection with it has reached a satisfactory standard. This is an instalment of a reform which has on previous occasions been advocated in The Hospital, though we should have been glad to see it pressed further to its logical conclusion—namely, that all candidates at their Final Examination shall be examined in ophthalmology. It is, further, a step in the right direction, that of increasing the relative importance of the subjects for the final examinations as against that of the preliminary sciences, which have for some years past been allotted an excessive proportion of the student's available time. If all teachers and all schools could be entirely trusted to work the regulation now adopted in the spirit as well as in the letter, no doubt the further reform here suggested would be unnecessary. But experience shows that this is not likely to happen; and, further, the procedure of the examining bodies is subject to inspection by the nominees of the General Medical Council, whereas the granting of certificates by the Deans of the various medical schools is not thus supervised: and medical school authorities are only human.

### DIFFICULTIES IN THE MEDICAL SCHOOL.

The Anatomical Committee of London is drawing the attention of public authorities to the fact that in every medical school in London there is a serious shortage of subjects for dissection, and asking them to improve the supply of unclaimed bodies. During recent years many Boards of Guardians have discontinued the practice of sending the bodies of persons dying in their institutions, who have no relations, to the medical schools, with the result that this shortage has arisen. As they adhere to this procedure, it is exceedingly difficult to see how the shortage is to be surmounted, but possibly the Anatomical Committee might, by a careful explanation to the Guardians, induce some Boards to alter their decision. But it is considered doubtful.

### THE WAR IN AFGHANISTAN: OUR STAFF MAN ON FIELD SERVICE.

The Afghan War has caught me, and I am on field service again. It has been rather unfortunate for us that we should be caught as soon as we got out almost. We were getting our staff of servants together and collecting furniture for the really nice bungalow I had secured when this war came without any warning and I was ordered on active service with the First Division. All women were ordered out of the Station, as there was good chance of a battle at our very gates. I was through the Khyber with the Division, and we were fighting the Afghans on May 11. We pushed them out of British-Indian territory, and followed them up into Afghanistan and fought a fiercer battle on May 16 and May 17. We had over 400 casualties. We passed 380 wounded down in the shortest possible space of time, " a record for India," the Divisional General says. We are the advanced division. We have got something under twenty miles into Afghanistan. We have done very well, and have surprised the Afghans by the celerity of our advance. We took Dakka unopposed, practically, so disorganised were the Afghan forces by the battle of May 11. We fought and fought successfully on the 16th and 17th to hold the position we had gained. Because it is not possible to take such vast quantities of artillery, nor is it of such heavy calibre, about this rough and mountainous land as was used in France, the battles are more spectacular. At Dakka I was able to watch the enemy advancing over the plain and scrambling into the heights that commanded us and to see our men and their men fighting and falling. It was an exciting time. The heat is very great, and the more trying because there are no trees to give shade, and a terrible amount of dust. So far sickness has not been very great. The future, who can forecast. It makes me glad to have had a chance to see something of Afghanistan. Few Englishmen get into the country, and fewer get out again. So far it is all mountainous, the hills very rough and barren. In small valleys are very small scraps of cultivation. Along the Kabul River is a considerable extent where the hills fall back far enough to give space, but I do not think that in any part we have seen the area of crops along the Kabul River more than a mile wide. Jelalabad may show more. But, though Afghanistan is as big as the old Germany with Belgium, Holland, and Denmark thrown in, the population it supports is only some 6,500,000. So much of the land is barren mountain or hopeless desert. We get sniped at at night in camp. Last night I heard a bullet sing past the tent I and others were sitting in, but the chances of a shot, shot at a venture, hitting one are very few. There are no shells to speak of.

### THE LATE EDITH CAVELL.

An old and dear American friend, Dr. Henry M. Hurd, Emeritus Professor of Psychiatry, Johns Hopkins University, Baltimore, has sent us the following tribute:—" I write to acknowledge with sincere thanks the reception of a copy of The Hospital of May 24 containing the very touching account of the honours paid to Edith Cavell. I congratulate you upon the skill and good feeling which have been shown in the tributes to her. The number is a triumph of sensible and judicious literary work. No one can realise who does not talk to people in this country how deeply the outrage against humanity and decency has brought a feeling of horror to all right thinking people in the United States in respect to the German mind. How can such standards of propriety, duty and decency be tolerated any longer? England must be much worn out by the long war and the great delay in making Peace. She has done so nobly in the trials of the last four years, and I am sure that she will come forth in Peace with great honour and renew her youth."

### THE IRISH ASYLUM STRIKE.

There is no administrator who will not sympathise with Dr. Harvey, medical superintendent of the County Tipperary Asylum at Clonmel, where the attendants have gone on strike. His most difficult task is to feed the patients, about twenty of whom have escaped, in a few cases to be " arrested " by the police. To prevent further escapes police are now stationed about the asylum. According to report, Dr. Harvey has had to tackle the situation almost unaided. Last week the managing committee, to whom he had telegraphed, failed to arrive, and in the absence of its members it was impossible for him to arrange a settlement. Help was no less difficult to find, and the staff was reduced to a few attendants who remained to look after the epileptic and violent cases. Matters have been made no easier by the mass of inquiries received from relations of the patients, and one cannot but say that if those concerned had been as eager to offer their services as they were to bombard Dr. Harvey with their fears a better public spirit would have been shown. A strike of a hospital staff is a very serious proceeding, and, to put it mildly, every expedient should be tried before it is decided on. Only so can the attendants hope to justify such conduct towards the sick. The inquiry, which must necessarily follow, will no doubt show on whom the responsibility must lie. But whatever their grievances the attendants will be hard put to it to justify their reinstatement. Hospital strikes cannot be allowed to occur.

### THE FARNBOROUGH INCIDENT.

At the time of writing Colonel McPherson's report is not to hand on the alleged assault on a blind patient at the Ontario Military Hospital, Orpington, by a civilian in Farnborough. Two military patients, one of whom was blind, state that on being asked to leave the bar of an inn at Farnborough, they were hustled by a civilian who later struck the blind soldier. The repetition of the story at the hospital naturally created excitement, and a number of convalescent soldiers visited Farnborough the next day in search of the offending civilian. The excitement then spread to the town, to which the commanding officer was summoned with Captain Hethrington, to find between forty and fifty crippled soldiers and a collection of police. The men formed up, and after replying to Colonel McPherson's questions, were driven back to hospital in ambulances. No rioting occurred, and the men were promised that the matter should be investigated.

### HOSPITAL NEWS IN LOCAL PAPERS.

With the return of medical officers to their hospitals on release from military duty, the experience gained in surgical or anæsthetic technique is beginning to be practised in the smaller hospitals. We notice with pleasure that the secretaries of provincial hospitals are recording the introduction of their refinements in their local papers, and to that extent interesting the public in the reputation of their institution. The Warneford General Hospital, at Leamington Spa, is an example, where the successful introduction of a recent method of anæsthesia has been made public. It is an instance of the welcome which awaits any interesting facts from a hospital in the newspapers. The secretary of Warneford Hospital, Mr. C. R. W. Oppen, should remember this and develop the art of making the hospital interesting, which does more than many appeals to secure steady support.

### CIVIL SERVICE AWARDS IN BERMONDSEY.

We are glad to say that the Bermondsey Borough Council has adopted the latest Civil Service Award for all its officials, and we trust that their example will soon be followed by other sanitary authorities both in London and the Provinces. No class of men has suffered more from the great increase of commodities and income tax than officials, and we think the Bermondsey Borough Council are to be congratulated on the recognition of the difficulties under which the officials have had to work during the last five years.

### THE FIRST MUNICIPAL GENERAL HOSPITAL.

Now that St. Luke's Hospital, Bradford, much enlarged during its occupation by the military, has reverted to the Bradford Board of Guardians, Mr. R. S. Dawson, the chairman, has been negotiating with the Health Committee and the representatives of the Ministry of Health. The guardians offer to let St. Luke's Hospital to the corporation on similar conditions to those on which it was let to the Government during the war, except that the guardians will not diminish their responsibility for the sick poor. The plan is for the corporation to run the hospital, and for the guardians to pay for the patients whom they find it necessary to send. If the plan succeeds Bradford would possess the first municipal general hospital, an addition, though an important addition, to the hospitals for infectious cases, sanatoria, maternity, and child welfare institutions already administered by the Health Committee. Since it is expected that the Ministry of Health will quickly include institutional treatment in the benefits of the Insurance Act, the already admitted need for more hospital beds will become even more apparent. St. Luke's possesses 1,200. The Royal Infirmary at present 200 only. If the plan is adopted, the war will be the date to which we shall look as the moment of change, for the adaptation of Poor-Law institutions to the use of military patients has paved the way for municipal general hospitals.

### PAYING PATIENTS IN POOR-LAW INFIRMARIES.

The Stoke-upon-Trent Board of Guardians has appointed a sub-committee to consider the advice of the medical officers if a number of beds at the Union hospital can be set aside at a reasonable fee for the benefit of the thousand cases which are on the waiting-list of the North Staffordshire Infirmary. Dr. W. Hind and Dr. Newton apparently put forward the proposal at a conference, and emphasised the need of those who could not afford the fees of a nursing home. The guardians, when appointing the sub-committee already referred to, favoured the plan which we hope can be successfully carried out. Mr. Beech pointed out how surprised many of the suggested patients would be at the beautiful institution, which a closer acquaintance would lead them to admire and not avoid. He believed that a new phase was being begun, which should be fostered and encouraged, for it would prove extremely beneficial. It is noteworthy that the scheme sprung from a desire to reduce the long waiting list at the infirmary, and we commend the goodwill of all parties to other voluntary hospitals and boards of guardians whose infirmaries are modern and properly staffed.

### A CLEVER LEAFLET.

The needs of the blind have a tireless friend in Sir Arthur Pearson, who has hit on rather a good appeal for the National Institute for the Blind. The covering letter short and sensible (and how difficult it is to make an appeal that!) It even contemplates the attitude of those Gallios who read such letters only to throw them away. The appeal itself enables the reader to test his own sight sufficiently to judge if it is "normal." The leaflet deserves a generous response, and we hope will receive it.

### THIS WEEK'S DRUG MARKET.

Business has been somewhat quieter during the past week, but the tone of the market still continues to be quite healthy, and there is a fair amount of inquiry which will probably lead to business in due course. The general downward movement of prices is still in check, and it is doubtful if it will recommence again on anything like the same scale that was witnessed a few months ago. Several drugs, however, have moved downwards. Aspirin, for instance, by no means appears to have reached its lowest level, and there are cheap offers on the market. Barbitone is also cheaper. English pressers of almond oil have reduced their quotations. Caffeine is offered at lower prices, as also is benzoic acid. Cocaine still has a downward price tendency, and the price of citric acid is not so firmly maintained. On the other hand, camphor still moves upwards in price, and the value of menthol is firmly maintained.

## Figures and Tables

**Figure f1:**